# The Chernobyl Disaster and Beyond: Implications of the Sendai Framework for Disaster Risk Reduction 2015–2030

**DOI:** 10.1371/journal.pmed.1002017

**Published:** 2016-04-25

**Authors:** Amina Aitsi-Selmi, Virginia Murray

**Affiliations:** 1 Public Health England, London, United Kingdom; 2 Epidemiology and Public Health Department, University College London, London, United Kingdom; 3 UNISDR Scientific & Technical Advisory Group, Geneva, Switzerland

## Abstract

On the 30th anniversary of the Chernobyl disaster, Amina Aitsi-Selmi and Virginia Murray reflect on the importance of disaster preparedness.

Thirty years have passed since the terrible Chernobyl nuclear power plant accident in what was then the Soviet Union. A nuclear reactor exploded on April 26, 1986, giving rise to a large plume of radioactive material. At the time, it was the most serious nuclear accident ever to occur [1]. The world held its collective breath in fear of radiation as the story of the accident and its impact in Russia and Europe slowly unfolded.

An intergovernmental review conducted 20 years after the incident by the Chernobyl Forum—comprised of an international team of more than 100 scientists—concluded that the impacts were less severe than originally predicted [2]. Despite the estimated 4,000 cases of thyroid cancer and about 4,000 deaths expected to be a result of the disaster, fewer than 50 deaths had been directly attributed to radiation from the disaster, almost all being in highly exposed rescue workers [2,3]. However, more recent reviews remind us that the long-term effects are still to be evaluated [4].

Among the recommendations of the Chernobyl Forum report was to address the lack of accurate information available to local populations on the health risks from the disaster itself as well as wider health risks, such as non-communicable diseases. The report also recommended taking into account and addressing socioeconomic challenges in the region [2]. Among the wider, cultural factors put forward as contributing to the Chernobyl disaster was the Soviet Union's isolation from the rest of the world and the lack of networks and personal contacts with scientists from other countries [5].

The 2011 Fukushima nuclear power plant disaster in Japan, triggered by a magnitude 9.0 earthquake and resulting tsunami, was a sobering reminder that even contemporary systems are vulnerable to natural hazards and complex in their interdependencies with natural and human factors [6]. Indeed, much work remains to be done to normalise a comprehensive, multidimensional approach to reducing disaster risk.

## United Nations Member States Adopt the Sendai Framework for Disaster Risk Reduction 2015–2030

Learning from the lessons of disparate disasters such as Hurricane Mitch in 1998 and the 2004 Indian Ocean Tsunami, the international community has broadened its approach, moving from a focus on response to including prevention, preparedness, and recovery and rehabilitation, and embracing multisectoral and multidisciplinary action that links with sustainable economic development and climate change [7,8].

The year 2015 brought the endorsement of a landmark UN agreement, the Sendai Framework for Disaster Risk Reduction (DRR) 2015–2030, which aims to reduce disaster losses in terms of lives, livelihoods, and health. It was adopted in March, 2015, by 187 UN member states. This agreement is an opportunity to strengthen international cooperation in science and technology and ensure that scientific knowledge is useful, usable, and used in emergencies.

The Sendai Framework puts unprecedented emphasis on the role of science in understanding and delivering risk reduction. It builds on its predecessor, the Hyogo Framework for Action 2005−2015: Building the Resilience of Nations and Communities to Disasters [9], and reflects shifts in scientific thinking over the last 20 years, with a powerful implication that disasters are not natural events against which human societies are powerless, but are the result of the interaction between hazards (natural and human-made), exposure levels, and pre-existing vulnerability.

Important recommendations of the Sendai Framework to the scientific community and its partners include improving the scientific and public understanding of risk and optimising the use of science for decision-making (Box 1). It makes more than 30 explicit references to health (compared to three in the Hyogo Framework), highlighting the importance of outbreaks and epidemics, chronic disease management, psychosocial interventions, and rehabilitation as part of disaster recovery and makes several references to the International Health Regulations [10]. The latter, if implemented properly, have the potential to reduce the risk of disasters such as the recent West African Ebola outbreak, which has been called “the definitive humanitarian disaster of our generation” [11]. Harking back to the slow speed with which communities were informed of the Chernobyl disaster, the Sendai Framework makes a recommendation to “invest in, develop, maintain and strengthen people-centred multi-hazard, multisectoral forecasting and early warning systems.”

**Box 1. Key Paragraph of Recommendations**

Key paragraph of recommendations to the scientific community for strengthening the evidence base and informing disaster risk reduction policy (para 25g, The Sendai Framework for Disaster Risk Reduction 2015–2030) [7]:

“Enhanced scientific and technical work on disaster risk reduction and its mobilization through the coordination of existing networks and scientific research institutions at all levels and all regions…to strengthen the evidence base in support of the implementation of this framework; promote scientific research of disaster risk patterns, causes and effects; disseminate risk information with the best use of geospatial information technology; provide guidance on methodologies and standards for risk assessments, disaster risk modelling and the use of data; identify research and technology gaps and set recommendations for research priority areas in disaster risk reduction; promote and support the availability and application of science and technology to decision-making; contribute to the update of the terminology on disaster risk reduction; and use post-disaster reviews as opportunities to enhance learning and public policy and disseminate studies.”

The Sendai Framework is a strong call to action for improving decision-making through a stronger science–policy–practice nexus with one expected outcome (“The substantial reduction of disaster risk and losses in lives, livelihoods and health…”), one goal (“Prevent new and reduce existing disaster risk through the implementation of integrated and inclusive…measures that prevent and reduce hazard exposure and vulnerability to disaster, increase preparedness for response and recovery, and thus strengthen resilience”), four priorities for action, and seven targets [7]. Some consider that reconnecting science with policy and practice is among the first tasks in implementing the Sendai Framework [12], particularly to support people in low- and middle-income countries and especially minority groups and women. A large body of research exists to support political and financial investment in the eradication and disruption of both the intergenerational transmission of poverty and the perpetuation of socioeconomic inequalities [13]. However, the Sendai Framework is a voluntary agreement, and its implementation will depend on political will, financing, and the imperative to collaborate across institutional and country boundaries, as well as the availability of data to monitor its targets [14].

One of the first implementation conferences was the Science and Technology Conference that took place in Geneva, Switzerland, on January 27–29, 2016, and brought together disaster risk reduction scientists, practitioners, and decision-makers from around the world. Important outcomes were to gain global agreement on a 15 year Road Map for the Implementation of the Sendai Framework [15], and the launch of a global Science and Technology Partnership to support the Road Map [16]. Only time will tell whether 2015 had its intended impact, and progress on the Sendai Framework objectives will be reviewed at the biannual Global Disaster Risk Reduction Platforms as we progress towards the 2030 goals. Recent devastating disasters resulting from historically unprecedented events, such as the 2011 floods in Thailand and Typhoon Haiyan in the Philippines in 2013, illustrate the challenge of increasing exposure to hazards (in this case, the predicted increased frequency and intensity of extreme weather events) alongside rising vulnerability and exposure through urbanisation and demographic change [17]. The importance of disaster preparedness can no longer be ignored.
